# Advancements in Imaging Technologies for the Diagnosis of Lung Cancer and Other Pulmonary Diseases

**DOI:** 10.3390/diagnostics15070826

**Published:** 2025-03-25

**Authors:** Alireza Nathani, H. Erhan Dincer

**Affiliations:** Section of Interventional Pulmonology, Division of Pulmonary, Allergy, Critical Care & Sleep Medicine, University of Minnesota, 420 Delaware St SE, MMC 276, Minneapolis, MN 55455, USA

**Keywords:** image technology, lung diseases, cancer

## Abstract

Advancements in imaging technologies have redefined pulmonary medicine, with increased diagnostic accuracy and improved clinical outcomes. This review discusses the evolving landscape of imaging advancements, including the pivotal role of low-dose computed tomography (CT) in lung cancer screening and the transformative impact of endobronchial ultrasound on lung cancer staging. Imaging techniques like high-resolution CT remain indispensable for the diagnosis and monitoring of parenchymal lung diseases. Positron emission tomography (PET) is increasingly being used for inflammatory conditions like sarcoidosis. In pleural diseases, thoracic ultrasound is essential in diagnosing and performing bedside procedures safely. Advanced modalities like ventilation scans have also been used to target persistent air leaks. This review emphasizes the importance of advancements in imaging technologies to the field of pulmonary medicine and underscores the continued innovation and integration of these advancements.

## 1. Introduction

Advancements in imaging technologies have profoundly transformed the field of medicine, particularly pulmonary medicine, where they have significantly enhanced the diagnosis and management of both benign and malignant conditions. Pulmonary medicine encompasses a diverse and complex array of diseases that often overlap in clinical presentation, preferred diagnostic tests, and, at times, even pathological findings. This overlap can present diagnostic challenges, even for the most experienced clinicians. As a result, the field necessitates a nuanced approach that integrates state-of-the-art imaging modalities with evolving procedural techniques. This review explores the advancements in imaging techniques for some of the most prevalent pulmonary diseases, focusing on lung cancer, parenchymal lung diseases, and pleural diseases.

## 2. Lung Cancer

Cigarette smoking is responsible for approximately 90% of lung cancer cases [1]. Recognizing the importance of early detection, the United States Preventive Services Task Force (USPSTF) updated its guidelines in 2021 to recommend lung cancer screening starting at age 50 for individuals with a 20-pack-year smoking history. Screening is advised to continue until the age of 80 but not beyond it [1].

Chest computed tomography (CT) has become a cornerstone in the evaluation of lung cancer, serving as a noninvasive tool for both screening and staging [2]. Among these, low-dose computed tomography (LDCT) has demonstrated its utility in reducing lung cancer mortality by up to 24%, making it an essential component of early detection strategies [3]. LDCT employs significantly less radiation than standard CT scans, allowing for safer repeated screenings in asymptomatic individuals who are at high risk. The effective dose of radiation was determined to be approximately 2 millisieverts (mSv) compared to an average effective dose of 7 mSv for a typical standard-dose chest CT in the National Lung Screening Trial (NSLT) [4].

The cost-effectiveness of LDCT lung screening, as evidenced by the NSLT, involves a delicate balance between the reduction in lung cancer mortality and the associated costs of screening, follow-up procedures, and treatment. This remains a complex issue influenced by numerous factors, and ongoing analyses aim to provide deeper insights into optimizing the benefits of LDCT screening while minimizing economic and clinical burdens.

To accurately diagnose and treat lung cancer, a pulmonologist or thoracic proceduralist must accurately stage or determine the extent of cancer in the body at the time of diagnosis. There are noninvasive methods to achieve this, including CT and positron emitted tomography (PET) CT, minimally invasive techniques including endobronchial ultrasound, and invasive techniques such as video-assisted thoracoscopic surgery. For the aim of this review, we will focus on the noninvasive and minimally invasive techniques.

CT scans are a crucial imaging modality in diagnosing lung diseases, particularly parenchymal diseases. As mentioned earlier, they are instrumental in lung cancer screening. There are solid and subsolid nodules. The focus of this review will be on solid nodules; however, it should be noted that subsolid nodules are a nonspecific radiologic finding. They can represent a benign or malignant disease. Pulmonary adenocarcinoma manifesting as a subsolid lesion is generally considered indolent and followed by CT surveillance [5]. Surgical intervention is suggested for nodules that exhibit growth with an increase in the solid component larger than 6 mm [6]. CT can also be helpful in determining the size and location of a tumor and detecting the presence of enlarged lymph nodes and metastases. CT imaging modality helps determine the extent of disease, which is helpful for treatment planning purposes. CT scans are often used in conjunction with other imaging modalities, such as PET. This form of imaging can provide a comprehensive evaluation of cancer spread. While CT scans are helpful in determining anatomical definitions, the metabolic nature of lung cancer is determined by PET scans. The technique for performing a PET scan is noninvasive. It involves injecting the patient with a radio-labeled tracer, such as 18F-fluorodeoxyglucose (FDG), and identifying where the tracer is absorbed in the body [7]. PET CT plays a crucial role in guiding biopsy decisions and identifying disease progression. For example, if we were to consider a patient who presents with a 2 cm right upper lobe nodule and enlarged right hilar lymph nodes, PET CT could assess metabolic activity in the right hilar lymph node to optimize the biopsy approach. If the hilar lymphadenopathy is PET-avid, sampling it over the primary nodule could reduce unnecessary procedures and risks. Second, if the PET reveals an incidental finding which was not expected, for example, a liver lesion suspicious of metastatic spread, the patient should have this safely biopsied to obtain the highest stage and most accurate diagnosis of their cancer.

**Figure 1:**
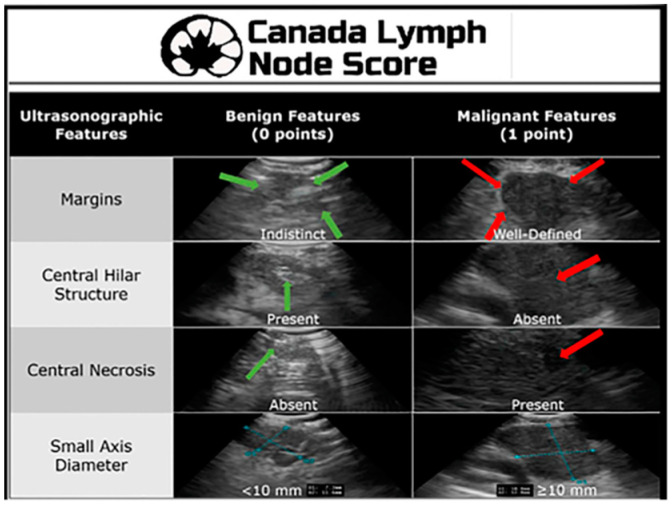
Canada Lymph Node Score. Reproduced from He et al., 2021 [10]. Reprinted from The Annals of Thoracic Surgery 115, 1456-1462 (2023), with permission from Elsevier.

Although the combination of a PET scan and a CT can be immensely helpful in determining location and potential stage of a malignancy and is considered to be better than CT or PET imaging alone, it still does not provide the clinician with a tissue diagnosis. Despite the high accuracy of PET-CT scans in detecting malignancy, the false-positive and false-negative rates (25% and 20%, respectively) cannot be ignored and require histological confirmation [8]. Endobronchial ultrasound with transbronchial needle aspiration (EBUS TBNA) is a technique that pulmonologists frequently use to obtain tissue for lung cancer diagnosis and staging. Historically, this procedure was performed blindly with a Wang needle in conjunction with CT anatomy to locate the lymph node. This technique had a poor yield range of 20–89%, depending on lymph node size, location, and operator experience [9]. Subsequently, a convex-probe EBUS was developed and first used in 2003. This technique allowed for real-time visualization of the lymph node under biopsy, changing the practice of mediastinal lymph node sampling [9]. A series of studies have shown that the diagnostic yield of EBUS is up to 91% [9]. The Canada Lymph Node Score (CLNS) uses four sonographic criteria to assess malignancy risk in lymph nodes during EBUS. The ultrasonic features assessed include the absence or presence of lymph node margins, the absence or presence of a central lymph node hilar structure, the absence or presence of central hilar necrosis, and the diameter of the lymph node (Figure 1). A higher CLNS correlates with an increased risk of malignancy, although a score of 0 can still show a malignancy rate of 16.9% [10]. Moreover, ultrasound elastography has emerged as a newer technique to aid in differentiating between benign and malignant lymph nodes by assessing their stiffness. The principle is based on the deformation of tissues in response to an applied force. Malignant tumor cells proliferate rapidly, causing stiffness and internal pressure. Malignant lymph nodes typically exhibit a homogenous hard elastographic pattern. The stiffness of a lymph node is imaged on a color map [11]. Alternatively, mediastinoscopy, video-assisted mediastinoscopic lymphadenectomy (VAMLA) and transcervical extended mediastinal lymphadenectomy (TEMLA) are surgical techniques that utilize a rigid mediastinoscope to perform excisional biopsy of mediastinal lymph nodes at different stations that cannot be reached by EBUS or in cases where more tissue is required for a definitive diagnosis [12].

Once a peripheral lung nodule or mass is identified on a CT, the next step is to obtain a tissue sample for diagnostic testing. Peripheral lung nodules have historically been approached with image guidance (e.g., CT) needle aspiration or core biopsy. With advancements in technology and expertise in interventional pulmonology, this approach is no longer the standard of care. There are a variety of tools that a pulmonary physician may use to help them perform bronchoscopy and biopsy of the peripheral lung nodule, and many of these use advanced imaging technology, such as robotic-assisted bronchoscopy (RAB), ultrasound, electromagnetic navigation (ENB), cone-beam CT, and optical coherence tomography (OCT).

**Figure 2:**
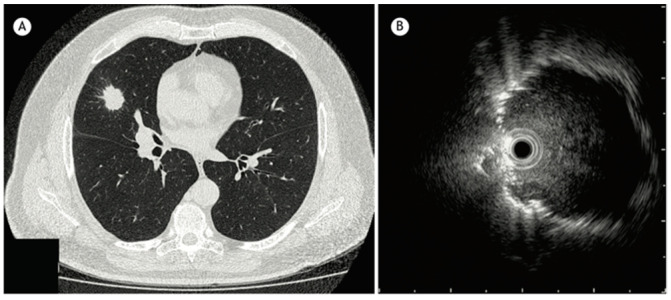
(**A**) CT chest showing a 2.5 cm nodule in the middle lobe. (**B**) Radial EBUS image reproduced from Jacomelli et al., 2015 [13] (Shared under the terms of the Creative Commons Attribution License, https://creativecommons.org/licenses/by/4.0/).

Radial endobronchial ultrasound (R-EBUS) is a diagnostic tool used in pulmonary medicine to visualize and evaluate lung lesions, particularly those not visualized by standard bronchoscopy. It involves the use of a rotating ultrasound probe (20-Mega Hertz) that is flexible and feeds through the working channel of a bronchoscope that emits ultrasound waves at 360 degrees to create detailed images of the lung tissue and surrounding structures (Figure 2). The probe is advanced into the tracheobronchial tree to reach the target lesion. The diagnostic yield of a transbronchial biopsy using a radial probe along with bronchoscopy is approximately 66.7% [13].

ENB is another tool pulmonologists use to help diagnose peripheral pulmonary lesions. ENB utilizes dedicated software to convert CT scan images into a multiplanar virtual bronchoscopy reconstruction of the patient’s airways. Once 3-D mapping is complete, a navigation tool is inserted into the patient’s airways, and the guide is tip-tracked with an electromagnetic field, which provides real-time imaging shown on the 3-D map. Once the tip-tracked tool reaches the target, samples can be obtained. Despite the advancement in technology, the diagnostic yield is similar to that of radial EBUS [14]. When both ENB and R-EBUS are combined, the diagnostic yield is up to 88%; however, these studies should be read carefully to discern selection bias, size, and location of the nodules [15].

Cone-beam CT (CBCT) is a type of CT that emits a cone-shaped X-ray beam, which acquires images over a large volume in a single scan. This is different from conventional CT, which uses a fan-shaped X-ray beam to acquire single-axial images. This type of technology has been used recently along with radial EBUS and robotic-assisted bronchoscopy to increase the diagnostic yield to 80%, while maintaining a high safety profile compared to CT-guided transthoracic biopsies. The pneumothorax rate using CBCT is approximately 2%, while the bleeding rate is 1% [16].

Endobronchial optical coherence tomography (EB-OCT) is a three-dimensional imaging technique that utilizes near-infrared light waves. These light waves have different reactive indices in various tissues, allowing for the system to generate defined images of the tissue structures. The OCT probe is flexible and inserted through a bronchoscope, like radial EBUS. The probe then scans images of the bronchial wall, including the different mucosal layers and cartilage. The images generated are in real time and can display both longitudinal and transverse sections of the tissue. Advantages of this type of imaging include the fact that it is free of radiation, noninvasive, and has the ability to provide high-resolution images. The yield of this particular imaging technology is similar to that of CBCT [17].

Robotic-assisted bronchoscopy has demonstrated a significant improvement in diagnostic yield for pulmonary nodules. A recent meta-analysis showed a sensitivity of 84.2% for malignant lesions. Several factors contribute to a higher diagnostic yield. These include a lesion larger than 2 cm, a concentric, radial EBUS signal, and a CT bronchus sign. The main benefit of robotic-assisted bronchoscopy is the precision for better navigation and stability during sampling, which is what separates it from other technology discussed. Using radial EBUS with robotic-assisted bronchoscopy also results in lower complication rates, such has hemorrhage and pneumothorax [18].

## 3. Parenchymal Lung and Airway Diseases

High-resolution CT (HRCT) is of vital importance to the clinician diagnosing and monitoring the progress of parenchymal lung diseases. Some of the key lung diseases where HRCT is particularly helpful include idiopathic pulmonary fibrosis, hypersensitivity pneumonitis, sarcoidosis, nonspecific interstitial pneumonia, organizing pneumonia, emphysema, and cystic lung diseases. HRCT can be carried out using two techniques: volumetric imaging and spaced axial imaging. The former technique captures the entire lung, which reduces sampling error and provides a comprehensive view of the lung. It is ideal for detecting subtle changes over time. The latter technique is acquired at intervals 0.5–2 cm intervals apart. This is performed to reduce radiation exposure. It is an adequate mode of imaging for many conditions; however, small abnormalities can be missed in addition to early changes in disease progression [19].

**Figure 3:**
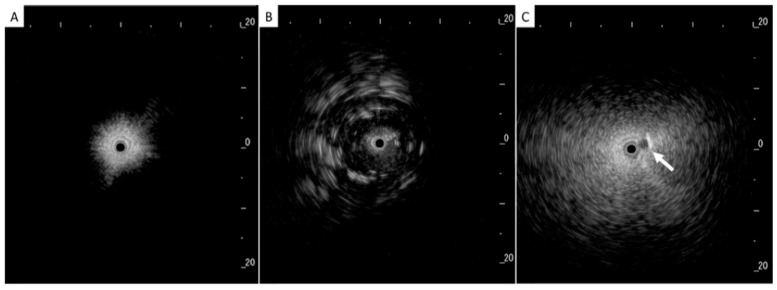
(**A**) Normal lung, (**B**) dense sign, and (**C**) blizzard sign. The white arrow is a pulmonary artery. Reproduced from Inomata et al., 2020 [22]. Shared in accordance with the Creative Commons Attribution Non Commercial (CC BY-NC 4.0) license, which permits others to distribute, remix, adapt, build upon this work non-commercially, and license their derivative works on different terms, provided the original work is properly cited, appropriate credit is given, any changes made indicated, and the use is non-commercial. See: http://creativecommons.org/licenses/by-nc/4.0/.

**Figure 4:**
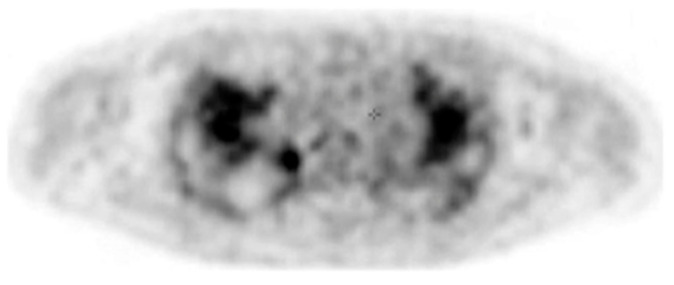
PET image at the thoracic level showing diffuse bilateral parenchymal FDG uptake. Reproduced from Mostard et al., 2011 [24]. Reprinted from Respiratory Medicine 105, 1917–1924 (2011) with permission from Elsevier.

**Figure 5:**
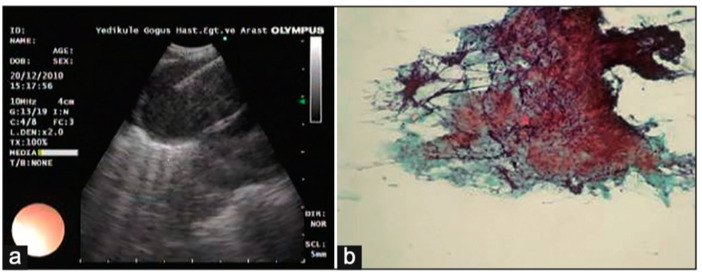
(**a**) Lymph node with granular appearance and (**b**) granular appearance associated with non-caseating granuloma on histopathology. Reproduced from Ozgul et al., 2014 [25]. Shared under the terms of the Creative Commons Attribution-Noncommercial-Share Alike 3.0. See: https://creativecommons.org/licenses/by-nc-sa/3.0/.

In addition to HRCT, there are several other imaging advancements very similar to the ones discussed in the lung cancer section that help in the diagnosis and monitoring of parenchymal lung diseases, including OCT, ultrasound, and PET. Earlier, we reviewed how OCT can be helpful for diagnosing lung nodules. Similarly, OCT offers minimally invasive high-resolution images for ILD diagnosis [20]. OCT has been shown to have sensitivity and specificity of 100% in detecting usual interstitial pneumonia patterns [21]. External validation and larger studies are required for clinical implementation. This could reduce reliance on surgical lung biopsy. Another form of imaging used includes ultrasound, which we have seen used for lung nodules as well. For parenchymal lung diseases, the pattern of abnormality is typically more diffuse. The radial ultrasound probe is inserted through a bronchoscope into the patient’s airways until it reaches the pleural surface. The probe is then slowly withdrawn from the pleura to the hilum, while examining the characteristics on the ultrasound image. This method helps determine a better location to biopsy. A dense sign correlates with a higher pathological confidence than a blizzard sign (Figure 3). Moreover, radial EBUS helps clinicians choose appropriate biopsy sites for cryobiopsy and identify blood vessels close to lesions, which should be avoided when a biopsy is performed. Minoru et al. demonstrated high-quality lung specimens with dense signs on radial EBUS while decreasing the bleeding risk [22]. Sarcoidosis is another common multisystemic disease characterized by the formation of non-caseating granulomas in various organ systems. Establishing the presence of inflammatory activity is crucial for diagnosis and the monitoring of treatment. One of the most common organs affected by sarcoidosis include the lungs. This condition affects the lungs in more than 90% of patients [23]. Sarcoidosis can affect the parenchyma, the mediastinal and hilar lymph nodes, or both. Increased FDG uptake in the pulmonary parenchyma is associated with inflammatory activity, presumably inflammatory cells, including activated macrophages, lymphocytes, and neutrophils, and, possibly, disease severity (Figure 4). One cannot solely rely on a PET scan for diagnosis and must use other forms of diagnosis, including history, physical examination, and serologic markers. It was found by Mostard et al. that 20% PET scans are positive without serological signs of inflammatory activity. PET can add value to the assessment of sarcoidosis and other inflammatory lung diseases in patients with symptoms despite the absence of serologic inflammatory markers [24]. Other patients do not exhibit parenchymal disease and just have mediastinal and hilar lymphadenopathy. In addition to PET scans, which can show hypermetabolic uptake in lymph nodes, ultrasound with linear EBUS can be used to examine the lymph node in real time and obtain tissue samples. Characteristics that can be examined include lymph node size, shape, margin, echogenicity, central hilar structure, and granular appearance (Figure 5). Furthermore, EBUS TBNA has shown a sensitivity of 89–100% and a specificity of 94–96% in diagnosing sarcoidosis [25]. This procedure and imaging technique can be used for diagnosing other thoracic diseases that infiltrate the mediastinal and hilar lymph nodes, such as infectious causes like TB or fungal infections, other inflammatory diseases like hypersensitivity pneumonitis and vasculitis, and malignant diseases.

Chronic obstructive lung disease (COPD) is the third leading cause of mortality in the United States. Patients suffer from breathlessness due to hyperinflation and air trapping. An endobronchial valve is the guideline-supported treatment of choice for patients who exhibit significant air trapping and hyperinflation. To select candidates for bronchoscopic lung volume reduction, patients must undergo CT to evaluate the destruction score of the lung tissue. This is determined by a percentage of lung voxels with a density less than −910 Hounsfield units [26]. The CT also helps clinicians assess the heterogeneity index by comparing destruction scores between target and ipsilateral lobes. Equally important is the assessment of fissure completeness, and HRCT scans are used to evaluate the completeness of the fissures between lobes. A complete fissure indicates minimal or no collateral ventilation. All of these factors, in addition to patient clinical history, pulmonary function test, six-minute walk test, arterial blood gas, and echocardiogram data, help select a patient who would appear to benefit from this procedure.

In recent years, a novel “optical biopsy” technique called confocal laser endomicroscopy (CLE) has been introduced in pulmonary medicine, providing high-resolution and real-time microscopic assessment of tissue architecture [27]. This technology, although it is in infancy, has the potential to diagnose pulmonary malignancies, ILD, pneumonia, lung transplant rejection, and COPD without ex vivo conventional sampling. CLE provides high-resolution images of lung tissues. It uses low-power laser light at 488 nm to illuminate the tissue. There are two forms of CLE: probe-based and needle-based CLE. The latter is for accessing deeper tissues. While promising, this technology is not part of routine clinical practice.

## 4. Pleural Disease

Pleural diseases encompass a wide spectrum of conditions affecting the pleural cavity. These include diseases that cause pleural effusion, pneumothorax, and persistent air leaks (PALs). This group of diseases poses challenges to the clinician addressing them. Accurate evaluation of these conditions relies heavily on advanced imaging modalities and procedural techniques. Two forms of imaging that we routinely employ are ultrasound and ventilation/perfusion scans.

**Figure 6:**
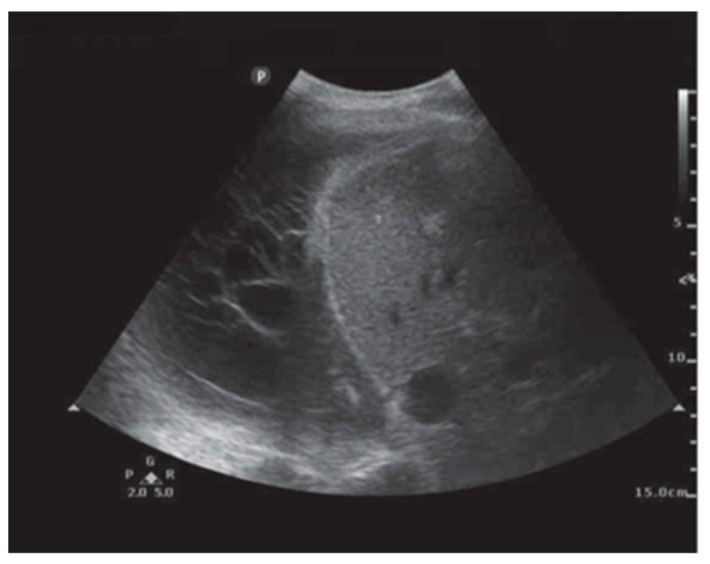
Pleural ultrasound revealing septations. Reproduced from Hassan et al., 2020 [28]. Shared under the terms of the Creative Commons Attribution Non-Commercial Licence 4.0. See: https://creativecommons.org/licenses/by-nc/4.0/.

Ultrasound has emerged as an essential tool for the assessment of pleural abnormalities and the therapeutic drainage of air or fluid. Ultrasound machines use transducers that emit ultrasound waves with specific frequencies and wavelengths. These waves penetrate tissues and reflect back to create a 2-D image. The time taken for waves to return determines the distance of structures. Air and bone have high attenuation coefficients, making it very difficult to form images. Conversely, fluid has a very low attenuation coefficient, which is great for pleural effusions. A transducer has a specific frequency attached to it. A high frequency (7.5–12 MHz) is used to image shallow structures like the chest wall and the parietal pleura. A low-frequency transducer (2–5 MHz) is used for deeper structures, like pleural effusions and the diaphragm. Most of the time, an ultrasound of the chest is performed by a clinician interested in answering a diagnostic query. The ultrasound beam needs to pass through the rib spaces, which can be challenging, and this is why patient positioning is crucial to optimizing image acquisition. There are three different imaging modes a clinician may use when performing an exam. The B-mode (brightness mode) is the most common mode used. It provides a grayscale image and allows for real-time procedural guidance. The M-mode (motion mode) provides a one-dimensional image showing movement over time. This mode is useful for pneumothorax evaluation and assessing pleural apposition presence or absence. Finally, the Doppler mode adds color images to the grayscale image so that the clinician may identity blood vessels, blood flow direction, or other moving fluids. This mode is particularly helpful when performing a thoracentesis and identifying intercostal vessels to avoid. Thoracic ultrasound has several advantages. It is mobile, so the clinician can bring this technology to the patient. Because it allows for real-time invasive procedural guidance, an improved safety profile is associated with pleural procedures. Finally, thoracic ultrasound is more sensitive than CT in detecting pleural fluid characteristics, such as septations (Figure 6) [28]. Knowing that an effusion is multiseptated is important for guiding therapeutic decisions. Real-time thoracic ultrasound can allow for the precise placement of chest drains into the largest locule, optimizing treatment and outcomes.

A pneumothorax is a condition where air enters the pleural space, leading to lung collapse, and it can be a medical emergency [29]. Ultrasound allows for the rapid diagnosis of a pneumothorax, often faster than an X-ray or a chest CT scan. This speed is crucial in situations where timely intervention can be lifesaving. There are several characteristics and patterns to recognize when performing an ultrasound exam for pneumothorax. It is important to define A and B lines. A lines are horizontal repetitive artifacts that originate from the pleural line. B lines are vertical, often described as comet-tail artifacts which originate from the pleural line. They indicate the presence of an interstitial syndrome, such as pulmonary edema. The first step in assessing a patient for a pneumothorax is to identity the pleural line. An ultrasound probe (usually a high-frequency probe) is held like a pen, allowing for stability and minimal fatigue for the operator. The BLUE protocol separates the chest into zones 1–3. Zone 1 is the anterior chest, zone 2 is the lateral chest wall, and zone 3 is the posterolateral chest wall. Furthermore, each of these zones is divided into upper and lower halves, resulting in a total of six areas of investigation. For a pneumothorax, the focus is on the anterior chest wall [30]. The next question to ask in this situation is whether there is lung sliding present, which is typically described as ants marching across the A line. If this is present, then a pneumothorax is ruled out. There are other reasons why a patient may not have lung sliding in addition to a pneumothorax, so this by itself does not rule in a pneumothorax. The next step is to look for a lung pulse. A lung pulse is a rhythmic movement of the pleural line that corresponds to the cardiac cycle. If this is not identified, the next step is to assess for vertical artifacts (B lines). If this is also absent, a pneumothorax is very likely. A lung point is the most specific sign for a pneumothorax. It refers to the location on the chest wall where lung sliding and the absence of lung sliding coexist.

**Figure 7:**
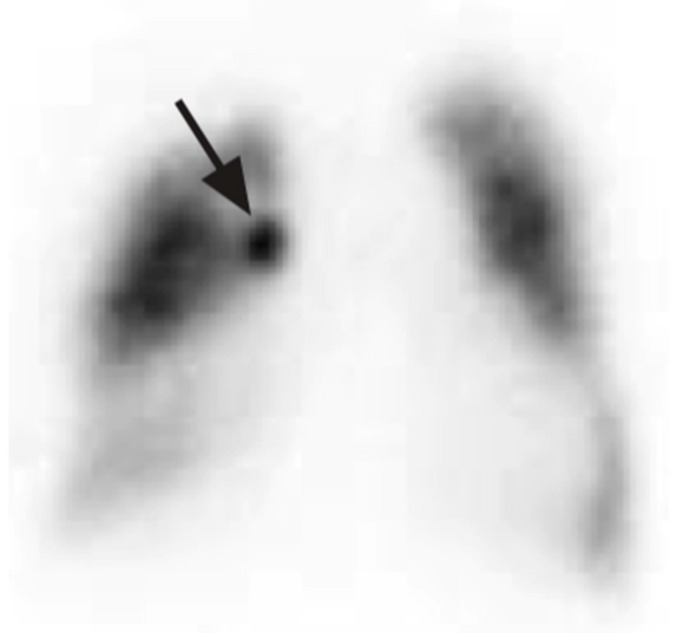
SPECT scan showing focal area of accumulation of 81 m-Krypton in the right upper lobe (arrow). Reproduced from Y.E. Ong et al., 2006 [31]. Reproduced with permission of the © ERS 2025.

One of the complications of pneumothorax and drainage is persistent air leakage, which carries high mortality and morbidity. The diagnosis of an air leak is often straightforward and can be made based on clinical findings showing subcutaneous emphysema, symptoms, and imaging studies showing pneumothorax or pneumomediastinum. Locating the air leak can be challenging but is the most important step in treating PAL, as early localization and treatment decrease mortality. Ventilation scans localize an air leak using a radioactive tracer. This is inhaled by the patient and allows for the visualization of airflow within the lungs using imaging techniques like single-photon emission computed tomography (SPECT). The tracer accumulates at the site of the leak, highlighting the specific area where air is escaping (Figure 7) [31]. Nakanishi et al. has published a method whereby 3-D cine CT is utilized with sequential dynamic 320-multidetector CT images, creating a 3-D movie after normal saline instillation via a chest tube into the pleural space [32]. Pulmonary scintigraphy using 99 m Tc-albumin has also been studied to locate the PAL site. This technique relies on colloid fog inhalation and the accumulation of the radiotracer in the bronchopleural fistula site [33].

## 5. Conclusions

Advancements in imaging technology have transformed the landscape of pulmonary medicine, offering clinicians the tools to improve their diagnostic capabilities and patient outcomes. While the imaging modalities and innovations discussed in this paper have addressed many diagnostic challenges, they also underscore the importance of continued technological advancement and accessibility.
